# Are there two disjunct episignatures for KMT2B-related disease?

**DOI:** 10.1093/braincomms/fcae383

**Published:** 2024-12-09

**Authors:** Konrad Oexle

**Affiliations:** Neurogenetic Systems Analysis Group, Institute of Neurogenomics, Helmholtz Munich, D-85764 Neuherberg, Germany; Institute of Human Genetics, Klinikum Rechts der Isar, Technische Universität München, D-81675 München, Germany

In an article published in *Brain Communications*, Monfrini *et al*.^1^ have proclaimed a specific DNA methylation profile at 175 CpG sites, which they derived from 8 individuals with heterozygous *KMT2B* missense variants and adult-onset dystonia or non-dystonic phenotypes. Since these individuals had probability scores close to 0 (≤0.016) when analysed by the conventional *KMT2B* episignature, such a disjunct methylation profile in them would not only be relevant for nosology and diagnostics. It would also impact the pathomechanistic concepts of *KMT2B*-associated disease, indicating a separate epigenetic mode of pathogenic action for a subset of *KMT2B* variants.

However, the analysis performed by Monfrini *et al*.^1^ has problematic aspects: (i) Three of the four variants observed in the affected individuals are predicted to be likely benign by recent prediction tools, i.e. Revel,^2^ MutScore^3^ and AlphaMissense,^4^ with prediction scores below 0.396, 0.150 and 0.125, respectively. Only one variant (p.R1078C), present in three of the eight individuals, has intermediate to high prediction scores, i.e. 0.505, 0.488 and 0.964, respectively. (ii) Despite this low prior probability, the DNA methylation sites of the specific profile have not been selected using a stringent genome-wide significance threshold. Instead, Monfrini *et al*.^1^ used the more relaxed and therefore error-prone false discovery rate correction of *P*-values. (iii) Most questionably, the proclaimed methylation profile was not tested and confirmed in an independent set of affected individuals, nor has any such validation been published since. Therefore, it remains well possible that in the eight training individuals, a contingent effect independent of *KMT2B* has driven their DNA methylation aberration.

Using the conventional episignature, allelic series for *KMT2B* have been detected, with low to intermediate probability scores in adult-onset or non-dystonic carriers of likely pathogenic missense variants.^5,6^ This is at odds with the report by Monfrini *et al*.^1^ on *KMT2B* missense variant carriers with adult-onset dystonia having a specific methylation profile that is entirely disjunct from the conventional episignature. In order to avoid unnecessary confusion in the field, it would be most important to know whether Monfrini *et al*.^1^ have replicated their finding in an independent set of patients.

## Data Availability

Data sharing is not applicable to this article as no new data were created or analysed.
